# Antioxidant Biomaterials in Cutaneous Wound Healing and Tissue Regeneration: A Critical Review

**DOI:** 10.3390/antiox12040787

**Published:** 2023-03-23

**Authors:** Nur Izzah Md Fadilah, Shou Jin Phang, Nurkhuzaiah Kamaruzaman, Atiqah Salleh, Mazlan Zawani, Arka Sanyal, Manira Maarof, Mh Busra Fauzi

**Affiliations:** 1 Centre for Tissue Engineering and Regenerative Medicine, Faculty of Medicine, Universiti Kebangsaan Malaysia, Kuala Lumpur 56000, Malaysia; 2 Department of Biomedical Science, Faculty of Medicine, Universiti Malaya, Kuala Lumpur 50603, Malaysia; 3 Department of Food Sciences, Faculty of Science and Technology, Universiti Kebangsaan Malaysia, Bangi 43600, Malaysia; 4 Department of Biotechnology, KIIT University, Bhubaneswar 751024, India

**Keywords:** antioxidant, biomaterials, wound healing, oxidative stress, delivery system

## Abstract

Natural-based biomaterials play an important role in developing new products for medical applications, primarily in cutaneous injuries. A large panel of biomaterials with antioxidant properties has revealed an advancement in supporting and expediting tissue regeneration. However, their low bioavailability in preventing cellular oxidative stress through the delivery system limits their therapeutic activity at the injury site. The integration of antioxidant compounds in the implanted biomaterial should be able to maintain their antioxidant activity while facilitating skin tissue recovery. This review summarises the recent literature that reported the role of natural antioxidant-incorporated biomaterials in promoting skin wound healing and tissue regeneration, which is supported by evidence from in vitro, in vivo, and clinical studies. Antioxidant-based therapies for wound healing have shown promising evidence in numerous animal studies, even though clinical studies remain very limited. We also described the underlying mechanism of reactive oxygen species (ROS) generation and provided a comprehensive review of ROS-scavenging biomaterials found in the literature in the last six years.

## 1. Introduction

Skin is the largest protective organ in the human body. It is constantly exposed to the external environment, which makes them vulnerable to potential insults. Skin injuries can cause severe problems such as the loss of body fluids, metabolism and immune system disorders, and life-threatening infections [1,2]. Briefly, the human skin is comprised of three main layers, including the epidermis, dermis, and subcutaneous layer. These layers vary significantly in terms of their anatomy and function [3]. Generally, the type of wound can be characterised based on the degree of damaged skin layers and the healing duration [4]. Superficial wounds are those in which only the epidermal layer is damaged. In contrast, partial-thickness wounds are those in which the epidermal and dermal layers, including hair follicles, blood vessels, and sweat glands, are damaged. Meanwhile, wounds with extensive tissue damage that involve the deeper layer of subcutaneous tissues are known as full-thickness wounds [5,6]. Tissue impairment caused by traumatic injuries, accidents, and other pathologies or diseases is now a common issue worldwide [7,8]. Wound healing involves a highly sophisticated and dynamic process to restore the integrity of the wounded area. Thus, the healing process might be delayed without proper wound care and rapid treatment. Some prerequisite requirements for successful wound healing include microenvironmental conditions, such as moisture, pH, and oxygen concentrations [9]. However, a chronic wound that exhibits delayed healing is often stalled at the inflammatory or proliferative phase. For example, chronic wounds in diabetic patients are often characterised by the hyperglycemia microenvironment that results in chronic inflammation and oxidative stress. Furthermore, several external factors, including wound infections, scab formation, and lack of oxygen supply at the wound site, and internal factors, such as vascular diseases and diabetes, may also lead to wound healing impairment [10]. Hence, new therapeutic targets and more effective treatment strategies must be identified in response to the above challenges.

Recently, there has been a paradigm shift in developing products intended for wound management through biomaterial engineering, which combines expertise from interdisciplinary areas of tissue engineering, regenerative medicine, materials science, molecular biology, and chemistry [11]. In medical terminology, a biomaterial is any natural or synthetic material that includes polymer or metal intended for interaction with living tissues. They are designed for better interaction with the biological systems, which can be used in various medical treatments, therapeutics, or diagnosis procedures [12]. Over the past decades, biomaterials have received extensive attention and played an integral and imperative role in wound healing applications, particularly in facilitating and expediting healing subsequent to a skin injury or tissue damage. The structural morphology of the biomaterials is one of the main criteria during scaffold fabrication for wound healing applications [13]. Generally, two-dimensional (2D) scaffolds can be achieved in the structure of films, membranes, and fibres, whereas three-dimensional (3D) networks with porous structures are available in the form of sponges, foams, and hydrogels. They can be applied in various wound types according to their morphology as wound dressings and bioactive tissue scaffolds [14,15]. These scaffolds are able to maintain a moist microenvironment for effective wound healing and accelerate the healing process by promoting cellular events such as proliferation and migration [16,17].

Following tissue injury, the inflammatory response plays a critical role in both normal and pathological healing. Our body’s innate immune system is activated immediately following an injury, thus initiating a rapid and localised inflammatory reaction. As a result, inflammatory cells are recruited from the circulation to react against the host tissue response [18,19]. However, upon the presence of persistent inflammation, excessive ROS are produced thus resulting in oxidative stress [20]. ROS are responsible for regulating the normal healing response and tissue repair process via different mechanisms [21]. Nonetheless, under certain pathological conditions, the level of ROS can exceed 500 µM in the inflammatory tissues, which is much higher compared to the normal tissue (1–15 µM) [22]. Considering that skin tissues are susceptible to oxidative stress, excessive ROS production can subsequently lead to protein dysfunction, abnormal cellular interaction, deoxyribonucleic acid (DNA)/ribonucleic acid (RNA) damage, and cell apoptosis [23]. For these reasons, antioxidants are proposed to overcome these limitations by inhibiting molecular oxidation and restoring the normal physiological level of ROS. However, these antioxidants are hindered by their low bioavailability and bioactivity when they are directly administered onto the wound. Therefore, by combining knowledge from biomaterials and skin tissue regeneration, intelligent biomaterials with antioxidant capabilities and functionalities to regulate ROS can be established. 

Antioxidant incorporation into biomaterials has a significant potential in designing therapeutics for wound healing. For example, in the study by Liu et al., the effect of an antioxidant-incorporated biomaterial, curcumin-loaded gelatin hydrogel, on skin healing was evaluated [24]. The in vitro results demonstrated that curcumin could enhance the process of wound healing by exerting its antioxidant property, inducing angiogenesis, promoting cell proliferation, and enhancing collagen formation at the wounded area. However, the direct delivery of pure curcumin into the body system has been hugely criticised due to its poor bioavailability. Interestingly, the in vivo results with diabetic mice revealed an improved efficacy upon the application of curcumin hydrogel when compared to the hydrogel only (control). Hence, this study proposes that the incorporation of antioxidants into biomaterial is a promising approach to improve the bioavailability and efficiency of the antioxidants. On the other hand, Bektas et al. investigated the effects of adding vitexin (C flavonoid glycoside ) to a chitosan-based gel to accelerate wound healing [25]. The incorporated vitexin-gel significantly improved healing activity in both in vitro and in vivo studies by enhancing cell proliferation and skin regeneration. Moreover, Pandey et al. fabricated a wound dressing with antioxidant activity for full-thickness wounds by preventing microbial infiltration, retaining wound moisture, and supporting cell proliferation [26]. Other than these studies, there are many compounds with antioxidant capacity that have been tested in vitro, in vivo, and in clinical trials, and the efficacy of these compounds in wound healing has been summarised by Comino-Sanz et al. [27]. 

Hence, developing biomaterials with antioxidant properties (in other words, incorporating antioxidant properties into a biomaterial) has become a growing effort and an important goal in improving the oxidative degradation of the biomaterial and the healing process of a wound. Antioxidant biomaterials have been used in various therapeutic applications, such as cosmetics [28], drug delivery systems [29], tissue engineering [30], and wound healing [31]. To achieve a sustained release, the antioxidant compounds can be either directly loaded or incorporated with the polymer chains [32]. Previous studies have reported a variety of bioactive materials and functional scaffolds that can enhance skin regeneration and damaged tissues based on their free radicals scavenging ability and antioxidant mechanisms. In this review, we present an update on the different types of responsive antioxidant biomaterials that have been implemented for wound repair. We also outlined the underlying mechanism of ROS generation and provided a comprehensive review of ROS-scavenging biomaterials that are specifically used in skin tissue engineering and wound regeneration.

## 2. Data Extraction Management

A literature search was conducted within six years of publications (2017–2022) through the platforms including PubMed, EBSCO host, Web of Science (WoS), Scopus, and Google Scholar. The search strategy used the terms ‘antioxidant’, ‘biomaterials’, ‘wound healing’, ‘oxidative stress’, and ‘delivery system’. Since this review is intended to cover the research of antioxidant compounds in combination with biomaterials for wound healing, we included any study that evaluated the effects of antioxidant biomaterials in the healing process (human studies). The exclusion criteria for this review would be all secondary literature and any original articles written and submitted in languages other than English.

## 3. Oxidative Stress

### 3.1. Wound Healing

Generally, the wound healing process comprises four chronologically overlapping phases, namely blood clotting (haemostasis), inflammation, proliferation, and remodeling [33,34]. Haemostasis is initiated immediately when a wound is induced and involves the initial formation of a platelet plug around the site of injury. This platelet plug is subsequently reinforced by a fibrin network formed through the blood clotting cascade, thus resulting in a stable blood clot to prevent excessive bleeding [33]. Following that, neutrophils and monocytes are recruited to the wound site, in which the monocytes differentiate into mature macrophages and regulate the inflammatory phase [33]. These immune cells produce pro-inflammatory cytokines and ROS to protect the body from pathogenic infection and breakdown damaged tissue (debris) at the wound site through a process known as autolytic debridement [35]. Under normal circumstances, the inflammatory cytokines, enzymes, and ROS are restored to basal levels within a few days after a wound is induced. Subsequently, keratinocytes and fibroblasts begin to proliferate and migrate towards the open wound area from the wound edge, whereas angiogenesis takes place to provide nutrients and oxygen to the wound bed via the newly developed vascular network [33,34]. Lastly, the extracellular matrix is rebuilt by forming collagen and other matrix proteins, to compensate for the lost dermis and forming granulation tissues [34]. Altogether, these processes resulted in complete wound healing. However, several factors, such as bacterial infection, necrosis, and hyperglycaemic microenvironment, can all lead to delayed wound healing. In certain cases, e.g., diabetic foot ulcer (DFU), all these factors might present in the same wound and result in severe wound impairment.

### 3.2. Importance of Redox Regulation in Normal Wound Healing

**Figure 1:**
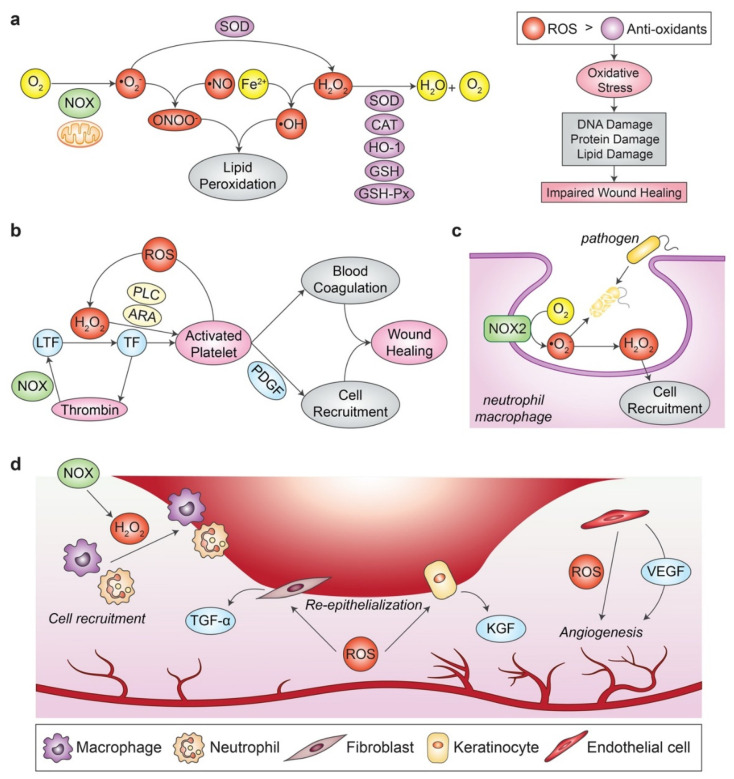
ROS and their role in regulating the process of normal wound healing. (**a**) •O_2_^−^ can be derived from O_2_ via the activity of NOX enzyme or the mitochondrial electron transport chain. Next, •O_2_^−^ can either form ONOO^-^ by reacting with •NO, or they can be converted into H_2_O_2_ via SOD activity. H_2_O_2_ can further react with Fe^2+^ to generate •OH, which combines with ONOO^−^ and causes lipid peroxidation. Hence, excessive H_2_O_2_ are normally eliminated by antioxidants like SOD, CAT, HO-1, GSH, GSH-Px by a reduction reaction into oxygen and water molecule. (**b**) LTF is activated by H_2_O_2_, which leads to thrombin synthesis and platelet activation. The generated thrombin in turn enhances ROS generation via the NOX enzyme, leading to a thrombogenic cycle via ROS-dependent signalling. The activated platelets promote blood coagulation to prevent excessive blood loss and recruit inflammatory cells by secreting PDGF, which altogether results in wound healing. (**c**) Potential pathogens that invade the wound bed are eliminated via the “respiratory burst” event in phagocytes like neutrophil or macrophage. These cells exhibit an upregulated NOX2 expression upon wound induction, which produces ROS as a weapon to kill the pathogens. (**d**) H_2_O_2_ produced from the NOX enzyme helps in inflammatory cells recruitment to the wound site. ROS production also enhances the re-epithelialisation process by promoting the TGF-α and KGF production in fibroblasts and keratinocytes, respectively. ROS also promote angiogenesis by the activation of endothelial cells via VEGF signalling. *ARA: arachidonic acid; CAT: catalase; Fe^2+^: ferrous ion; GSH: glutathione; GSH-Px: glutathione peroxidase; HO-1: heme oxygenase-1; H_2_O_2_: hydrogen peroxide; KGF: keratinocyte growth factor; LTF: latent tissue factor; •NO: nitric oxide radicals; NOX: nicotinamide adenine dinucleotide phosphate (NADPH) oxidase; O_2_: molecular oxygen; •O_2_^−^: superoxide anion; •OH: hydroxyl radical; ONOO^−^: peroxynitrite ion; PDGF: platelet-derived growth factor; PLC: phospholipase C; ROS: reactive oxygen species; SOD: superoxide dismutase; TF: tissue factor; TGF-α: transforming growth factor-alpha; VEGF: vascular endothelial growth factor. The idea of the figure is adapted from* [49,50,51].

ROS is the umbrella term for radical derivatives of the molecular oxygen (O_2_). The examples of ROS include, but are not limited to free radicals, such as superoxide anion (•O_2_^−^), hydroxyl radical (•OH), and non-radicals like hydrogen peroxide (H_2_O_2_) (Figure 1a) [35]. Considering most metabolic processes in the human body including wound healing require O_2_, the generation of ROS is therefore inevitable. Generally, cellular ROS in the human body is mainly produced by the membrane-bound nicotinamide adenine dinucleotide phosphate (NADPH) oxidase (NOX) enzyme complex and also the mitochondrial electron transport chain [36,37]. Although biological oxidants such as ROS are frequently depicted to possess detrimental effects that can impair wound healing, appropriate levels of these oxidants have been shown to be essential in maintaining normal body functions like wound healing [35]. In fact, ROS has been suggested to be involved in the multiple phases of the healing cascade, including haemostasis, re-epithelialization, and angiogenesis [35,38].

In response to wound injury, vasoconstriction and blood coagulation cascade occur, which are both mediated by ROS [39]. For example, the latent tissue factor is activated by H_2_O_2_, which in turn promotes thrombin synthesis (Figure 1b). The generated thrombin then enhances ROS generation via NOX, leading to signaling cascades that mediate a thrombogenic cycle via ROS-dependent signaling. Besides, platelet aggregation and recruitment to the wound site are associated with H_2_O_2_ synthesis via the arachidonic acid (ARA) metabolism and phospholipase C (PLC) pathway, which helps in preventing excessive blood loss. Following that, phagocytes such as neutrophils and macrophages initiate a “respiratory burst” or “oxidative burst” by secreting ROS via NOX as a weapon to eliminate potential pathogenic substances that invade the exposed wound (Figure 1c) [40,41]. NOX2 is abundantly expressed in the plasma membrane of the phagocytes, such as neutrophils and macrophages, and their expression is rapidly upregulated upon wound induction [42]. The chemotaxis of these inflammatory cells is also shown to be driven in the presence of H_2_O_2_ at low concentrations. In addition, ROS production enhances the re-epithelialisation process of fibroblasts and keratinocytes [35]. Evidently, transforming growth factor-alpha (TGF-α) and keratinocyte growth factor (KGF) have been shown to be induced by ROS in fibroblasts and keratinocytes, respectively (Figure 1d). Next, vascularisation of the wound bed is also dependent on ROS signaling as reports demonstrated that impairment in the NOX activity hinders the efficiency of the wound healing response. Low levels of H_2_O_2_ were also found to enhance the angiogenesis phase during wound healing [43].

Despite physiological levels of ROS being beneficial towards wound healing in a timely manner, excessive production of these molecules is detrimental towards a normal wound healing process. Thus, our body possesses an antioxidant defense system that can detect abnormal oxidant levels and react accordingly in order to balance the oxidants level and restore redox homeostasis. For example, enzymatic antioxidants, such as superoxide dismutase (SOD), catalase (CAT), and glutathione peroxidase (GSH-Px), as well as non-enzymatic antioxidants, such as heme oxygenase 1 (HO-1) and glutathione (GSH), are essential towards providing an antioxidant effect [35,44]. Besides that, small molecules of antioxidants, such as vitamin C and polyphenolic compounds, can also act synergistically with the endogenous defense mechanisms to maintain redox homeostasis. In addition, the nuclear factor erythroid 2-related factor 2 (Nrf2) plays a vital role in the antioxidant signaling pathway as a master regulator [45,46]. In the presence of oxidative stress, Nrf2 translocates into the nucleus from the cytoplasm and binds to the antioxidant response element (ARE) [47]. As a result, the transcription of the antioxidant enzymes, including SOD, CAT, and GSH-Px, and other antioxidants, such as HO-1 and GSH, are promoted, thus restoring the ROS level back to the basal level [42,46]. Intriguingly, the signal transduction and activation of Nrf2 have been recently proposed to be contributed by the phosphorylated Akt and adenosine monophosphate-activated protein kinase (AMPK) [48]. Taken together, these key molecular targets should be emphasised in generating antioxidant molecules with the aim to reduce the oxidative stress in certain pathological conditions.

### 3.3. Impact of Oxidative Stress in Chronic Wound

**Figure 2:**
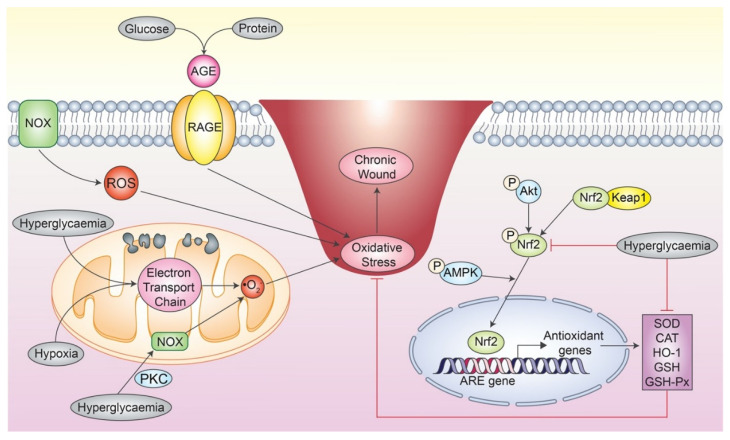
Oxidative stress underlying a chronic wound. Excessive ROS are produced via the mitochondrial electron transport chain under hyperglycaemia and hypoxic condition. Hyperglycaemia can also activate mitochondrial NOX activity via PKC. Next, the formation of AGE from the reaction between glucose and protein binds to its receptor, RAGE, which induces signalling that leads to oxidative stress. Besides promoting the formation of oxidants, hyperglycaemia also inhibits the Nrf2 signalling, which leads to downregulated expression of antioxidants. Altogether, the persistent redox imbalance leads to severe oxidative stress and results in a chronic wound. *AMPK: adenosine monophosphate-activated protein kinase; AGE: advanced glycation end product; ARE: antioxidant response element; CAT: catalase; GSH: glutathione; GSH-Px: glutathione peroxidase; HO-1: heme oxygenase-1; Keap1: Kelch-like ECH-associated protein 1; NOX: nicotinamide adenine dinucleotide phosphate (NADPH) oxidase; •O_2_^−^: superoxide anion; Nrf2: nuclear factor erythroid 2-related factor 2; PKC: protein kinase C; RAGE: AGE receptor; ROS: reactive oxygen species; SOD: superoxide dismutase. The idea of the figure is adapted from* [54,55,56].

Although the human body is well equipped with an antioxidant system, certain stimuli underlying some pathological conditions can produce excessive oxidant production, thus resulting in persistent oxidative stress and impairs normal wound healing progress. For example, tissue hypoxia is tightly associated with impaired wound healing due to oxidative stress [37]. Under hypoxic condition, excessive ROS are generated via the mitochondrial electron transport chain, which leads to oxidative stress and impaired wound healing [37]. Next, DFU is another classic example of a chronic non-healing wound [34]. The hyperglycemic environment underlying a DFU wound significantly promotes the generation of oxidants such as ROS (Figure 2) [44]. For instance, excessive glucose in the oxidative phosphorylation process in mitochondria produces excessive •O_2_^−^ as a by-product [42]. Besides, the mitochondrial NOX system is also activated by hyperglycemia via protein kinase C (PKC) stimulation, which also produces excessive free radicals [42]. Next, the production of advanced glycation end products (AGEs) can also contribute toward oxidative stress underlying the wound by several mechanisms. 

The binding of AGEs to their receptors (RAGEs) induces signaling cascades that increase intracellular ROS levels and ROS-generating enzyme expression, which impairs the anti-oxidative defense mechanisms [42]. Altogether, the excessive oxidants destroy the composition and structure of the extracellular matrix, causing oxidative damage and changes in gene expression, which then results in an impaired wound healing. Aside from generating excessive oxidants such as ROS, a chronic wound is also characterised by an impairment in the antioxidant defense mechanism, which intensifies the redox imbalance [45,46]. For example, the expression of SOD, CAT, GSH, and GSH-Px is found to be downregulated in diabetic patients [52,53]. Besides that, the Nrf2 signaling pathway is also dysregulated in a chronic wound. A downregulation of Nrf2 expression was observed in diabetic dermal fibroblasts, resulting in the decreased expression of downstream antioxidant enzymes [45]. Moreover, the wound healing rate of an Nrf2^−/−^ streptozotocin-induced diabetes mouse model was severely impaired compared to the Nrf2^+/+^ mouse model [46]. Similarly, the perilesional skin tissue of DFU patients also exhibited significantly higher Nrf2 expression compared to the normal patient, hence suggesting that DFU patients are under more severe oxidative stress that requires higher activation of the Nrf2 signaling pathway [46]. 

Different molecular targets can be identified by understanding the formation of oxidative stress and the antioxidant defense mechanisms to activate antioxidant signaling and inhibit the formation of oxidants. Hence, efforts to design biomaterials with antioxidant properties that can achieve redox balance are being focused, specifically to resolve chronic wound healing.

## 4. Natural Antioxidants

Antioxidants are defined as compounds that are present in relatively low concentrations and can prevent or inhibit oxidation [57]. Antioxidants can stabilise, deactivate, or scavenge free radicals that can harm cells. Generally, antioxidants can be grouped into natural antioxidants and synthetic antioxidants. Natural antioxidants are obtained from natural sources that are widely distributed in food and plants. These natural antioxidants from plant materials are mainly polyphenols, carotenoids, and vitamins [57,58]. They may occur in all parts of the plants, such as fruits, vegetables, nuts, seeds, leaves, roots, and barks [59]. These naturally occurring antioxidants display a wide range of biological activities and great nutritional values along with their primary antioxidative mechanisms. Besides, they are safe to consume and do not have any side effects. 

**Figure 3:**
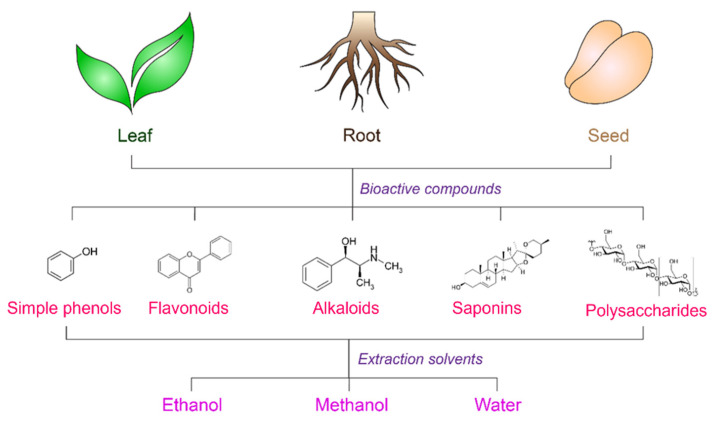
The selected plant parts, bioactive structures, and primary extractions of antioxidants are involved in wound healing. The idea of the figure is adapted from [57].

In recent years, extensive studies have been conducted on the topic of managing and treating wounds with plant-derived products. However, in this paper, we will only review recent papers published in the last six years on the potential use of selected plant parts (leaves, fruits, and seeds) that possess biological activity for wound healing. The leaf, fruit, and seed of plants are among the most promising sources of antioxidants. Leaf is one of the main sources of antioxidants because of its rapid growth and abundance. Fruits and seeds are also regarded as valuable sources of antioxidant potency because of their phytochemical characteristics and nutritional contents, such as fibres, vitamins, and micronutrients [60]. Here, the different sources of natural antioxidants from plant origins, their bioactive compounds, extraction methods, and biological activities that display evidence in wound healing are presented in Figure 3 and Table 1, respectively.

Phenolic compounds are the main antioxidant substances that promote wound healing (Table 1). They are the most abundant secondary metabolites produced by the plants. Based on their chemical structures, phenolic compounds can be classified into several subgroups. As identified by the majority of the studies presented here, these subgroups include phenolic acids, flavonoids, tannins, coumarins, lignans, quinones, and curcuminoids [61,62,63,64,65]. Among all the phenolic compounds described in this paper, flavonoids are the most abundantly used compound. These naturally occurring antioxidants display a wide range of biological activities, such as antioxidant, antibacterial, and anti-inflammatory properties, which qualifies their use as a biomaterial component for wound healing.

### 4.1. Extraction Methods

Given the biological effects exerted by these natural antioxidants, the extraction phase is the most important stage in plant-based antioxidant derivation. Various extraction parameters, including solvent type and concentration, temperature, duration, and pH, greatly influence the effectiveness of the extraction phase. The solvent, however, is the factor that has an enormous impact [57]. Antioxidants from plants have been extracted using a number of solvents. The chemical makeup and polarity of the extracted chemicals should be considered when choosing a solvent. Most substances in the chosen plant parts are hydrophilic phenolics and flavonoids. Hence, extraction processes typically utilise polar and medium-polar solvents, such as water, ethanol, methanol, and their aqueous mixes to obtain the best yield [66,67]. 

According to the literature in Table 1, the most commonly used extraction method was solvent extraction with alcohol and water. This solvent extraction method is typically known as conventional extraction, which primarily employs a hot water bath and Soxhlet extraction. Water is a universal solvent and most phenolic compounds are water-soluble. In addition, aqueous (water) extraction is also non-toxic and biocompatible with heat-sensitive compounds. In contrast, alcohol extraction is more effective in penetrating the cellular membrane and extracting the intercellular ingredients from plant material, thus resulting in higher activity of alcohol extracts compared to aqueous extracts [68,69]. However, some drawbacks were identified, including the time, costs, and the relatively large amounts of organic solvents that are required [70]. Due to each component’s unique chemical and physical features, there is no standard method for extracting natural antioxidants. Nonetheless, conventional extraction was often preferred because of its simplicity and low cost compared to the non-conventional extraction techniques using ultrasound, microwave, pressurised liquid, and supercritical fluids [57]. Taken together, the yield and quality of the extracts are highly dependent on the solvent and extraction method employed, as well as the process conditions. An efficient extraction is considered when the maximum amount of bioactive molecules can be extracted with the least amount of degradation and non-antioxidant components.

**Table 1:** Different sources of natural antioxidants from different parts of plant.

Part of Plant	Compound(s)	Source(s)	Extraction Method	Biological Properties	Reference
Leaves	Saponins	*Algerian urtica dioica* (neetle)	Methanolic extract	Antibacterial and antioxidant	[71]
	Marrubin, phenol and flavonoid	*Marrubium vulgare*	Methanolic extract; Microwave assisted solvent extraction (MASE)	Antihypertensive, analgesic, anti-inflammatory, hypoglycaemic, vasodilator, antidiabetic, and antibacterial	[61]
	Flavanoid (quercetin-3-O-rutinoside and catechin)	*Zizyphus lotus*	Methanolic extract	Antidiabetic, sedative and hypoglycaemic	[72]
	Phenol and flavonoid	*Trigonella foenum-graecum* (Fenugreek), *Cassia acutifolia* (Senna) and *Rhazya stricta* (Harmal)	Ethanolic extract	Antioxidant, anti-lipoxygenase and anticancer	[73]
	Tannin, saponin, flavonoid	*Sarasinula marginata*	Ethanolic extract	Antioxidant, responsible for slower release profile process	[74]
	Flavonoids, terpenoids and phenolic compounds	*Melia azedarach*	Water extract	Antibacterial, antidiabetic, and antioxidant	[75]
	Phenolic (caffeoylquinic acids)	*Dittrichia viscosa*	Ethanolic extract	Antiradical and antioxidant	[76]
	Retinol and alpha tocopherol	Rosemary and clove	Dried plants	Antioxidant and flavouring agent	[77]
	Vitamins, amino acids and anthraquinones, glucomannan	Aloe vera	Commercially available	Anti-inflammatory, antioxidant, antibacterial, control release, biocompatible, biomechanical stability, cell proliferation, attachment, re-epithelisation, angiogenesis, and high water uptake	[63,78,79,80,81]
	Epigallocatechin gallate (EGCG)	Green tea	Methane sulfonic acid and tetrahydrofuran	Anti-inflammation, and radical scavenger effects	[82]
	Alkaloids, polyphenols, phenolic acids, a range of flavonoids, and glusinolates	*Moringa oleifera*	water and methanol extract	Anti-inflammatory, antioxidant, and antimicrobial	[83,84]
	Coumarin compound named ostruthol	*Peucedanum ostruthium*	Ethanol and water extract	Anti-inflammatory, antibacterial	[64]
	Carotenoid, fucoxanthin, astaxanthin, and phenolic compounds such as tannins, flavonoid and phenolic acid	*Eucheuma cottonii* extract (red seaweed)	Water extract	Antioxidant, and high-gelling properties	[85]
Fruit	Alkaloids	Berberine	Analytical grade	Biocompatible, proliferation, and antibacterial property	[86]
	Lignin	Coconut husk	Ethanol and water extract	UV protective agent	[62]
	Flavonoids	*Capparis spinosa* fruit	Ethyl alcohol	Antioxidant	[87]
	Polyphenolic compounds, phenolic derivatives, flavonoids, and pectin, Vitamin C	*Cydonia oblonga* fruit (Quince fruit)	Ethanolic extract	Antibacterial, anti-inflammatory, anticancer, anti-bacteria and cardioprotective properties	[88]
Seed	Tannic acid and picotannic acid, pyrogallic acid, gratanotannic acid, resin, and mucilage, alkaloids	Pomegranate seed	Ethanolic extract	Antibacterial, anti-inflammatory, and antioxidant	[65]
	Fenugreek absolute (trigonelline and nicotinic acid)	*Trigonella* foenum graecum	Methanolic extract	Hypoglycaemic effect, hypocholesterolaemic activity and anti-ulcerogenic effects	[89]
	Silymarin	Milk thistle plant (*Silybum marianum*)	Commercially available	Antioxidant, antimicrobial	[90]
	Bixin	Seed	Ethanolic extract	Antioxidant, anti-inflammatory, and hypoglycaemic effects	[91]
	Fatty acid, tocopherol	Soybean	Analytical grade	Antibacterial, antioxidant, and anti-inflammatory	[92]

### 4.2. Advantages of Natural Antioxidants Properties on Wound Healing

To keep the body’s structure and functionality intact, tissue repair is a crucial mechanism. Free radicals, which can harm the healthy cells of tissues, can occasionally cause a delay in the body’s natural tissue repair process. In many instances, the presence of bioactive chemicals in the plant extract exhibits substantial antioxidant, antibacterial, and anti-inflammatory effects [64,65,71,76,86,88,92]. 

As free radical scavengers, antioxidants have been shown to effectively aid in tissue repair to reduce oxidative stress and maintain the free radical levels at a desired level. Low antioxidant levels can indicate high levels of free radicals in the body and vice versa. When the body has more free radicals than antioxidants, these excess radicals can attack the lipid, protein or DNA components leading to oxidative stress [93]. Furthermore, antibacterial agents are beneficial for wound infection control as they can delay the healing process. Due to the potentially toxic or harmful effects of many chemical antimicrobial agents, natural materials are preferred in the treatment of microbial infections [94,95]. Bioactive compounds extracted from different parts of plants efficiently promote different stages of wound healing. By exhibiting their potent antibacterial and antioxidant properties, these compounds can aid in the healing of various wounds [71,72,78,89,96,97]. 

Inflammation plays a crucial role in the innate immune system’s response to tissue damage or the creation of wounds under normal physiological conditions. Nevertheless, severe and uncontrolled inflammation can delay or limit the healing process [98,99]. Therefore, compounds with anti-inflammatory effects are also important and crucial in wound repair. 

## 5. Incorporation of Natural Antioxidants into Biomaterials

### 5.1. Types of 3D-Biomaterials

**Figure 4:**
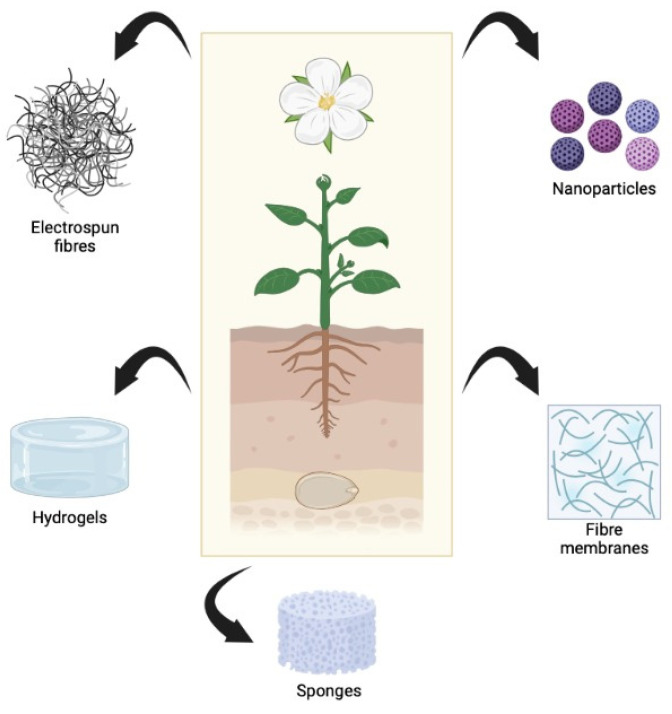
Different types of materials from plant-derived antioxidant compounds. The idea of the figure is adapted from [104].

As the field of tissue engineering and regenerative medicine is growing rapidly, research on biomaterials as therapeutical agents, especially in wound healing applications, has been extensively done to cater the increasing market demands [100,101]. Biomaterials, such as wound dressings or dermal templates, yield a promising future in the health industries as wound management is becoming one of the primary concerns. Regardless of whether the biomaterials are made from synthetic or natural sources, they must be biocompatible, non-immunogenic, possess suitable microstructural features to house cells, and facilitate cellular proliferation and tissue regeneration [102,103]. One of the mechanisms for biomaterials to enhance wound healing is the stimulation of a suboptimal inflammatory response, which is one of the most important phases in wound healing. However, it could be harmful if excessive inflammation occurs in which ROS is also overproduce. As a consequence, the microbalance between ROS concentration and antioxidant defense becomes disoriented, thus resulting in oxidative stress [20]. Therefore, it is beneficial to introduce antioxidant properties into the biomaterials. Antioxidant compounds derived from plants have great potential to be incorporated into different biomaterials, including electrospun fibres, sponges, nanoparticles, hydrogels, and fibre membranes (Figure 4). Meanwhile, Table 2 shows studies that are involved in incorporating antioxidant compounds in different types of biomaterials.

#### 5.1.1. Film/Membrane

Film or membrane dressings are one of the most common wound dressings that are available in the market [105,106]. These biomaterials are typically used in treating superficial or minor wounds due to their permeability towards vapors and oxygen, thus providing a moist environment for faster wound healing. They possess small pores that only allow the transmission of small molecules, such as oxygen, which are beneficial to prevent the invasion of microorganisms into the wound site. The flexibility of the biomaterials also helps treat a wound that is present in a hard-to-cover body area [107]. Additionally, film dressings are easy to fabricate, cost-effective, and, most importantly, thin and transparent to allow clinicians to monitor the progress of wound healing without removing the dressing [108]. However, this dressings category is hindered by their poor swelling properties, which hinder the absorption of blood and exudates. Hence, this limits their application for treating severe wounds with high exudates.

The incorporation of antioxidant compounds into films can enhance the functionalities of biomaterials and stabilizes them. For example, L. Colobatiu et al. have constructed novel bioactive chitosan films with great antioxidant activity, thus promoting wound contraction and rapid healing [109]. S. Paranhos et al. in a study reported that the presence of copaiba oil helps increase the chitosan membrane’s hydrophilicity and wettability effect with an increasing concentration of antioxidant compounds, which ultimately improves the film adsorption ability [110]. Furthermore, O. Dragonstin et al. have successfully developed a chitosan-derivative membrane that can facilitate wound healing of a burn wound in vivo. The membrane demonstrated rapid re-epithelialisation and faster wound healing compared to the control. Taken together, these studies suggest antioxidants as promising biomaterials to be used in wound healing applications [111]. 

Intriguingly, it was discovered that most antioxidant compounds also contain antibacterial effects. The antioxidant compound in the scaffold usually exerts its antibacterial properties by disrupting the bacterial membrane through the presence of highly hydrophobic compounds. For instance, M. Balasubramaniam et al. studied the antibacterial activity of novel biodegradable chitosan film incorporated with ferulic acid towards *Bacillus subtilis, Staphylococcus aureus*, and *Escherichia coli*. In the study, they concluded that the film showed excellent antibacterial activity comparable with vancomycin (positive control) [112]. Moreover, they also showed that the bacterial inhibition zone is more prominent in acidic scaffolds, which is attributed to the presence of phenolic hydroxyl groups that act as a proton changer, thus disrupting the proton-motive force and affecting the ATP pool. Meanwhile, Guilherme E. et al. reported that the bioactive gelatin films incorporated with clove essential oil exhibited good antibacterial activity toward *Staphylococcus aureus* and *Escherichia coli* [113]. The presence of phenolic compounds, such as eugenol and thymol, interferes with the phospholipid layer of the bacterial membrane, which increases the permeability and expulsion of bacterial cytoplasm [114,115]. 

Other than films, antioxidant-incorporated hydrogels are often discussed due to their high biocompatibility and low immunogenicity characteristics [21,116]. Notably, there are numerous differences between the functionality of a film dressing and a hydrogel. One of the most obvious differences is that film dressings serve as a barrier to the wound site while the hydrogel is implemented over the wound site. Therefore, hydrogels can deliver the antioxidant compound directly into the wound site, thus making them a promising candidate for wound healing applications [117]. 

#### 5.1.2. Hydrogels

In terms of bioactive molecule incorporation, hydrogels have been the primary option because of their simple processing requirement and the ability to encapsulate and release under controlled conditions. Thus, it is an ideal biomaterial to be used as a wound dressing compared to topical administration. These materials have excellent swelling properties, that enable them to absorb large amounts of wound exudates and blood, maintain a moist environment, and promote autolytic debridement [118,119]. Additionally, hydrogel dressings are ideal candidates for deep and irregularly shaped wounds [120]. Their highly tuneable properties allow them to be modified easily, thus making hydrogels suitable for application in multiple wound types [121]. For example, they can be formulated to enhance their responsiveness toward wound stimulation, modified to release specific mediators such as antibiotics with the goal of reducing infection, inflammatory reducers, and antioxidant compounds to reduce inflammation reactions. Nonetheless, the only drawback of hydrogel-based wound dressing is its poor mechanical stability in the swollen state. Notwithstanding this limitation, hydrogels are by far the most extensively investigated form of biomaterial scaffold for wound management.

Evidently, Park et al. have constructed a thermosensitive gallic acid-incorporated hydrogel intending to treat full-thickness skin loss [122]. The solid-gel transition of hydrogel in body temperature has been improved by adding gallic acid into the formulation. Gallic acid is capable of forming hydrogen bonding through inter/intramolecular interactions. This provides the biomaterial with a self-healing ability that could re-establish its native properties. The presence of gallic acid in the gelatin hydrogel showed a higher adhesive strength tested using a lap shear test with porcine skin due to the occupancy of pyrogallol, hydroxyl, and amine groups in the bioactive gelatin hydrogel. Nevertheless, the hydrogels showed excellent cell-scaffold compatibility as well as a rapid in vivo wound healing effect. On the other hand, F. Kong et al. have successfully integrated two bioactive components (sodium alginate and 5-hydroxymethylfurfural) into the hydrogel. They reported that the dual bioactive hydrogel has the highest water content, which is 90% higher compared to the native PVA hydrogel, whereas in vivo study of the hydrogels showed their capability to aid in vascularisation as well as enhancing ECM remodeling [123]. However, a different finding was reported by Y. Deng et al., whereby the pressure resistance of the bio-composite increased upon the addition of an antioxidant compound (flaxseed gum) [124]. Following that, the in vivo finding of the group demonstrated that the hydrogel has successfully aided in haemostasis and rapid wound healing. Considering that high mechanical strength is important to reduce the risk of biomaterial breaking during and post-implantation, so as not to limit the patient’s daily activity, the incorporation of antioxidant compounds is therefore indispensable. 

Furthermore, hydrogels loaded with antioxidant compounds also have been shown to confer resistance against bacterial growth. Singh et al. evaluated the bacterial permeability of the Carbopol-loaded gum acacia extract by leaving the hydrogel in an open environment for a month, which is then tested with the turbidity assay [125]. They reported no contamination within the hydrogel, which proposes that it possesses an effective antimicrobial barrier to prevent secondary infection at the wound site. P. Antezana et al. also reported that a composite containing cannabis *Sativa* oil extract has bactericidal properties that are highly susceptible to Gram-positive bacteria [126]. The antimicrobial mechanism of the cannabidiols is established by inhibiting the protein synthesis and interfering with the microbial membrane, which explains why the compound is more effective against Gram-positive bacteria that consist of only a single membrane layer. 

#### 5.1.3. Electrospinning Fibre

Electrospun fibres have enormous potential in the tissue engineering field due to their high porosity and unique topography that is easily modified due to the electrospinning process [127]. The molecular weight distribution, structure, viscosity, conductivity, and surface tension of the electrospinning solutions influenced the formation of electrospun fibre. Other parameters, such as the electric potential, the flow rate, and the distance of the capillary and collector can also influence the morphology and size of the fibre [128]. One of the most important characteristics of electrospun scaffolds is their high surface area-to-volume ratio. Indeed, this feature enhances the cell-scaffold interaction, which demonstrates an excellent biological activity to regulate cell function and tissue regeneration. It can also be engineered to deliver drugs, growth factors, or other bioactive molecules in order to promote tissue formation [129]. Nonetheless, the electrospinning technique for developing wound dressing has several limitations, including their high cost, time consumption, poor cell infiltration and migration due to the close arrangement of scaffold fibres, the toxicity of the residual solvent, and the low mechanical strength of dressings [130]. 

With the discovery of the electrospun scaffold incorporated with antioxidant compounds, Khandasamy et al. in a study incorporated a potent antioxidant, vitamin K3 carnosine peptide (VKC), into silk fibroin nanofibre. The nanofibre was presented in a thermally and structurally stable form due to the intermolecular forces of the VKC and silk fibroin nanofibre, which help them to sustain the native structural properties during the pre-application of the materials. The high mechanical properties of the bioactive electrospun fibres were influenced by the highly active site of bearing quinone and amide bond, which increase the affinity of fibroin microstructures. As a consequence, the fibrous biomaterials have successfully exhibited an excellent antibacterial activity towards both Gram-negative and Gram-positive bacteria and accelerated wound healing ability in a short period of time [131]. In addition, Augustine et al. have successfully created a 3,4-dihydroxyphenylalanine nanofibre that act as a stimulus to accelerate cell proliferation, migration as well as vascularisation in wound healing. The encapsulation was shown to be successful as the histological results revealed a ROS reduction in the wounded tissue, which proposes the preservation of antioxidant properties throughout the incorporation process [132].

#### 5.1.4. Sponge

Sponge-type biomaterials have been widely exploited for their ability to adsorb wound exudates as well as their effects on platelets aggregation. Many researchers have created multifunctional sponges incorporated with antioxidants, antibacterial agents, and growth factors to promote rapid wound healing. The freeze-drying technique is commonly used to fabricate sponge scaffolds as it involves a dehydration process by freezing the scaffolds and a sublimation process to remove the ice crystals. The main advantage of this fabrication technique is the elimination of solvent without biomaterial degradation and the formation of a high porosity structure [133]. Additionally, sponges have low density and therefore do not cause much discomfort when administered onto a wound. However, some limitations remain associated with this procedure, which include the long processing time and high energy consumption [134]. Cao et al. have successfully developed a multifunctional tannic acid-chitosan sponge with great potential in aiding haemostasis and rapid wound healing. Furthermore, the developed sponge also showed high antioxidant activity in various in vitro scavenging assays [135]. On the other hand, Tamer and colleagues have constructed a PVA-kaolin sponge that supports wound hemorrhage and has demonstrated outstanding DPPH and ABTS-scavenging activities [136]. Both sponges have excellent antioxidant activity and great haemostasis ability as the active compounds also act as haemostatic agents (kaolin and tannic acid).

#### 5.1.5. Nanomaterial/Particles

Nanomaterials for tissue regeneration can be developed under different structures including nanoparticles, nanocapsules, nanospheres, nanoemulsions, nanocarriers, and nanocolloids [137,138]. They have been widely studied and have significantly impacted the wound healing industries [139]. Nanoparticles can stimulate a variety of cellular and molecular processes that aid in the wound microenvironment through antimicrobial, anti-inflammatory, and angiogenic effects, which then potentially shift the environment from non-healing to healing [140]. With this regard, Sharma et al. have fabricated bimetallic nanoparticles incorporated with *Madhuca longifolia* seed, and they have demonstrated a promising wound healing potential of the nanomaterial based on the nanotech parameters [141]. In their study, a family of seven flavonoids was ascertained in the seed extract. For example, biofabricated nanomaterials, such as flavonoid-loaded gold, silver, and bimetallic nanoparticles, have been reported to enhance wound healing. These results suggest that the flavonoid contents loaded into the nanoparticles are being imparted with additional antioxidant properties. 

Another example of a nanoparticle that is commonly used in medical applications is gold (Au), which has become a promising approach for skin tissue engineering. Evidently, Cui et al. found that Au nanoparticles have unique physico-chemical properties, whereby they can scavenge ROS through their antioxidant activities [142]. A previous study by Lau et al. showed that Au nanoparticles accelerated the wound healing process by improving angiogenesis, proliferation of epithelial cells, and the formation of collagen [143]. The incorporation of bioactive molecules or nanoparticles into biomaterials with antioxidant properties is a growing strategy that is highly useful to enhance skin tissue regeneration. Moreover, the role of nanomaterials and scaffold-based tissue engineering approaches for accelerated wound healing via angiogenesis enhancement has been reviewed by Nosrati et al. [144]. The successful biofabrications are listed in Table 2.

**Table 2:** The available studies of biomaterials incorporated with antioxidant compounds.

Biomaterial	Antioxidant Component	Biological Effect	Study Design	Reference
Hydrogel	Gallic Acid	Antioxidant	In vitro and in vivo	[122]
	Chitosan Sodium alginate	Antioxidant, anti-inflammatory, and migration-promoting effects	In vitro and in vivo	[145]
	5-hydroxymethylfurfural	Anti-inflammation and anti-bacterial	In vitro and in vivo	[123]
	Curcumin	Anti-bacterial	In vitro and in vivo	[146,147]
	Vitamin E	Antioxidant	In vitro and in vivo	[148]
	Acacia gum	Non-haemolytic, antioxidant and mucoadhesive	In vitro and in vivo	[125]
	*Cannabis sativa*	Anti-inflammation, analgesic effects, antioxidant and anti-bacterial	In vitro	[126]
	Humic acid	Anti-inflammation and antioxidant	In vitro	[149]
	Propenoic acid	Antioxidant and anti-microbial	In vitro and in vivo	[150]
	Olive leaves	Antioxidant, anti-inflammation and anti-microbial	In vitro and ex vivo	[151]
	Tannic acid	Antioxidant and anti-bacterial	In vitro and in vivo	[152]
Nanogel	Lignin	Antioxidant	In vitro and in vivo	[62]
Films/Membrane	Quercetin	Antioxidant	In vitro and in vivo	[153]
	*Sarasinula marginata* extract	Antioxidant	In vitro and in vivo	[74]
	Ferulic acid	Antioxidant and anti-bacterial	In vitro	[154]
	*Plantago lanceolata*	Antioxidant	In vitro and in vivo	[109]
	*Tagates patula*	Antioxidant and anti-microbial	In vitro and in vivo	[111]
	*Symphytum officinale*			
	*Calendula officinalis*			
	*Geum urbanum*			
	Chitosan			
	Copaiba oil	Antioxidant	In vitro and in vivo	[110]
	Clove essential oil	Antioxidant and anti-microbial	In vitro	[113]
	Papain	Antioxidant and anti-microbial	In vitro	[155]
	Curcumin	Anti-inflammation, antioxidant, and anticancer activity	In vitro	[112]
Electrospun Fibre	Vitamin K3- Carnosine peptide	Antibacterial	In vitro and in vivo	[131]
	Honey	Antioxidant and anti-bacterial	In vitro	[156]
	Curcumin			
	Quercetin	Anti-bacterial	In vitro and in vivo	[157]
	Alkannin	Anti-inflammation, antioxidant, anti-mirobial and anti-tumor activity	In vitro	[158]
	Shikonin			
	Quinone	Antioxidant	In vitro	[159]
	Konjac glucomannan EGCG	Anti-bacterial	In vitro and in vivo	[160]
Nanoparticles	Catechol moiety	Antioxidant	In vitro and in vivo	[132]
	Seed of *Madhuca longifolia*	Antioxidant and anti-microbial	In vitro and in vivo	[141]
	Star anise	Anti-inflammation and antioxidant	In vitro	[161]
	*Gundelia tournefortii* leaf	Antioxidant, anti-fungal, and anti-bacterial	In vitro and in vivo	[162]
Aerogel	Wheat grass	Anti-microbial and angiogenic response	In vitro and in vivo	[163]
	*Hypericium perforatum* oil	Antioxidant and anti-microbial	In vitro	[164]
Bioactive glass	Curcumin	Antioxidant and anti-microbial	In vitro and in vivo	[165]

### 5.2. Antioxidants Activity of Integrated-Biomaterials

Considering the antioxidant capabilities of the antioxidants via different mechanisms as mentioned above, the antioxidants-embedded biomaterials have also been shown to stimulate free radical detoxification enzymes and aid in accelerating wound healing processes [166]. Furthermore, the antioxidant biomaterials’ ability to suppress chronic inflammation by inhibiting the nuclear factor-kappa B (NF-κB) transcription, IL-8 production, and LPS-induced inflammation also confers extra advantages over the basic biomaterials [167,168]. The biomaterials are used as the carrier, filler, or support system to ensure the delivery of antioxidants to the intended area and establish an optimum environment (good moisture and excess fluid absorption) to support the wound healing process.

**Figure 5:**
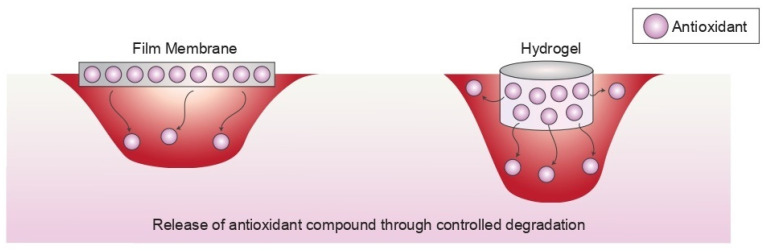
Control release antioxidant compounds from biomaterials. The idea of the figure is adapted from [169].

The release kinetics of antioxidant biomaterials are similar in different types of biomaterials, which involve a controlled release of the antioxidant compound through biomaterial degradation. Implanted biomaterials, such as hydrogels, sponges, and nanoparticles, have a more desired and wider release profile for the incorporated antioxidant compounds because they are integrated into the wound site. In contrast, the superficial biomaterials, such as films or membrane dressings, are only placed on top of the wound site as shown in Figure 5. 

In order to validate the antioxidant activity of these antioxidant-incorporated scaffolds, various antioxidant parameters can be performed. Among all, 2,2-diphenyl-1-picrylhydrazyl (DPPH), 2,2′-azino-bis (3-ethylbenzothiazoline-6-sulfonic acid) (ABTS), and dichloro-dihydro-fluorescein diacetate (DCFH-DA) assays are some of the most common antioxidant assays to be performed. Other antioxidant assays of the biomaterials for wound healing applications are also listed in Table 3.

#### 5.2.1. 2,2-Diphenyl-1-picrylhydrazyl (DPPH) Assay

**Figure 6:**
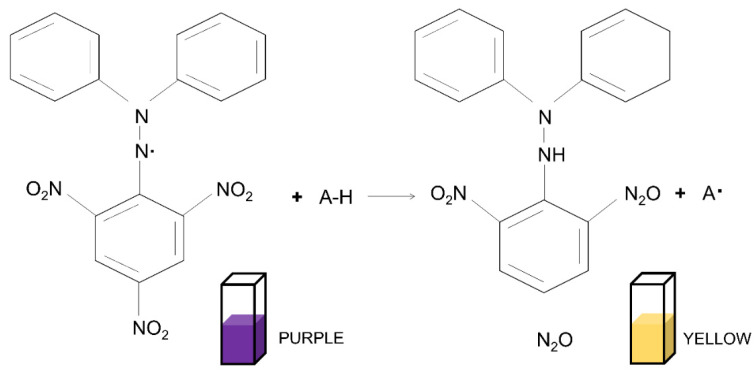
The reaction of DPPH assay when the antioxidant agent is present. The image is adapted from Marjoni and Zulfisa (2017) [172], licensed under Creative Commons Attribution License.

The determination of the scavenging ability of antioxidants in relation to it serves as the basis for the test. The unpaired electron on the nitrogen atom in DPPH is removed when the matching hydrazine receives an electron from an antioxidant in the form of a hydrogen atom. The delocalisation of the spare electron throughout the whole molecule in DPPH gives it the property of being a stable free radical. This property prevents the molecules from dimerising, which is a characteristic shared by a vast majority of other free radicals. The delocalisation is also responsible for the deep violet color, which exhibits an absorption in an ethanol solution at a wavelength of around 520 nm. When the DPPH solution is combined with anything that can donate a hydrogen atom, a reduced form is produced, but the solution’s characteristic violet color is lost in the process [171]. The overall reaction of the DPPH assay is described in Figure 6.

#### 5.2.2. 2,2′-Azino-bis (3-ethylbenzothiazoline-6-sulfonic Acid) (ABTS) Assay

**Figure 7:**
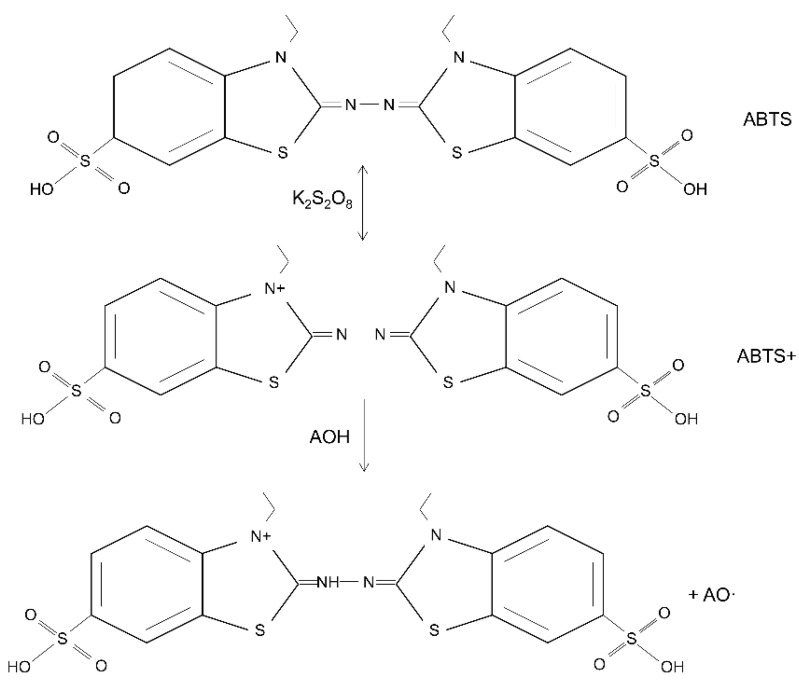
The interaction of ABTS assay when the antioxidant agent is present. The image is adapted from Bedlovicova et al. [174] and license under CC BY 4.0.

The ABTS test analyses the relative capacity of antioxidants to scavenge the ABTS that is formed in aqueous phase. Trolox, a water-soluble vitamin E analogue, is used as a standard in the ABTS assay. The ABTS is produced by subjecting the ABTS salt to a reaction in which a powerful oxidizing agent, such as potassium permanganate or potassium persulfate, is also present. The suppression of the distinctive long wave absorption spectra of the blue-green ABTS radical is used as a metric to measure the decrease of this radical caused by hydrogen-donating antioxidants. Trolox equivalent antioxidant capacity is the typical way that this approach is represented (TEAC). The procedure is quick and may be utilised in aqueous and organic solvent systems over a broad range of pH values. As a result of its high reproducibility as well as its ease of execution, it has received a lot of press coverage. On the other hand, the approach has not been associated with any biological consequences, and therefore its true significance to the antioxidant efficacy in living organisms is unclear [173]. The reaction of ABTS assay when in contact with antioxidant agents is described in Figure 7.

#### 5.2.3. 2′,7′-Dichlorodihydrofluorescein Diacetate (DCFH-DA) Assay

**Figure 8:**
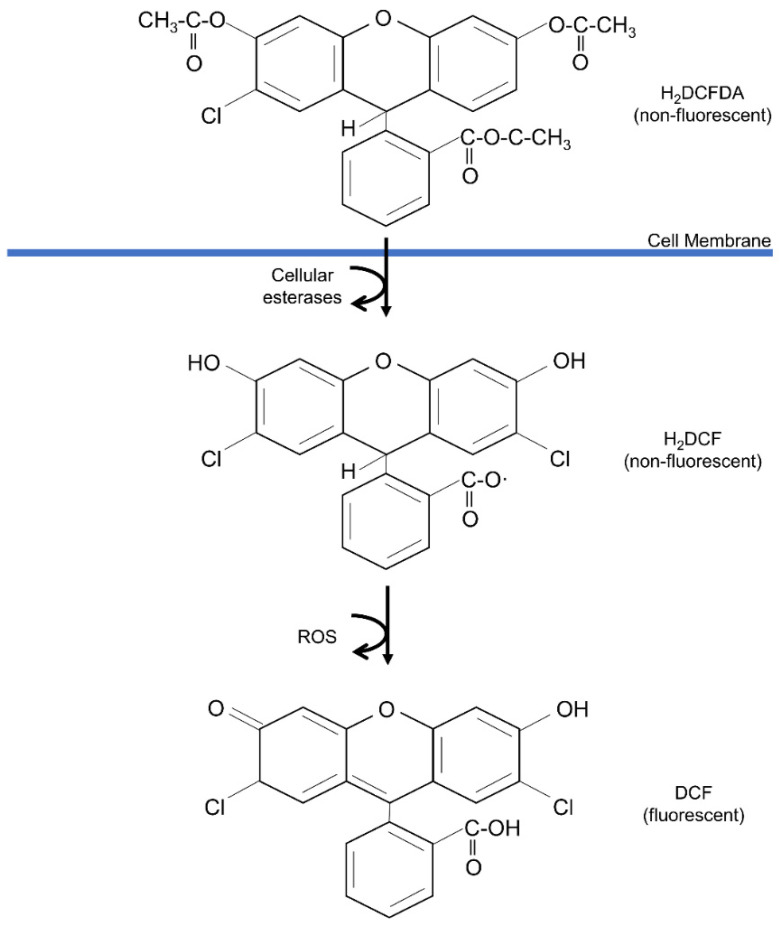
The chain reaction of DCFDA assay where the molecules become highly fluorescent after reacting with reactive oxygen species. The image is adapted from Nova et al. [176] and license under CC BY 4.0.

The DCFH-DA staining has been extensively used for total ROS detection, including nitrogen dioxide (•NO_2_) and hydroxyl radicals (•OH). The mechanism involved is the oxidation of DCFH to DCF by ROS, which emits green fluorescence at the wavelength of 485 nm (excitation) and 530 nm (emission), respectively [175]. The overall interaction of the DCFH-DA assay is illustrated in Figure 8.

## 6. Therapeutic Applications to Wound Healing

In essence, the antioxidant capabilities of bioactive compounds, together with the health-protecting properties of these compounds, might offer a promising technique in the field of tissue engineering and regenerative medicine. Many studies have demonstrated that various functionalised biomaterials are responsible for antioxidant properties. Antioxidant treatments have been administered to reduce the rate of organ deterioration that occurs ex vivo prior to transplantation. Currently, researchers are looking for alternative antioxidant buffers to extend the viable period of an ex vivo biological material. In a similar fashion, antioxidant supplies might protect the survival of ex vivo grafts, such as cornea and skin grafts, which in recent decades have been utilised at the level of good manufacturing practices (GMP) for clinical application [177,178]. As a result of the fact that all tissues share the same fundamental process to maintain a redox balance, the manipulation of oxidant species levels by antioxidants may be applied in a broad variety of contexts. The introduction of an antioxidant into tissue substitutes will, in essence, create an impact, the nature of which will be determined by the concentration of the antioxidant that is released and the interactions between the ECM components.

### 6.1. Pre-Clinical Models for Antioxidant Biomaterial Evaluation

#### 6.1.1. In Vitro Analysis (Cell-Based Studies)

Pre-clinical studies remain the gold standard to evaluate cytotoxicity, safety, and dose efficiency before proceeding to the next stage of clinical study. Pre-clinical or cell-based studies are widely used to screen and determine the effects of compounds or biomaterials on human cells. It is also often utilised to measure the binding receptors as well as signal transduction which express genes, cellular components, and organelle function monitoring. One of the cell-based methods is the MTT assay, a rapid colorimetric assay, which relies on the cleavage of the MTT (3-(4,5-dimethylthazolk-2-yl)-2,5-diphenyl tetrazolium bromide) tetrazolium ring by the living cells’ mitochondrial dehydrogenases. The number of viable cells can be estimated by measuring the intensity of the purple formazan formed [179,180]. Meanwhile, a scratch assay is utilised as the preclinical model for wound healing studies that aid in understanding the cellular response mechanism to initiate migration and cell-cell interactions [181]. Moreover, immunocytochemistry (ICC) has been widely used to analyse the protein expressions of the cells. ICC involves antibodies and antigens in which the enzyme-conjugated antibodies convert chromogen substrates to a colored precipitate at the reaction site through chromogenic detection [182]. Different studies that involve the pre-clinical or cell-based study are tabulated in Table 4. 

The in vitro model of skin study mostly consists of cells that play important roles in the wound healing process such as keratinocytes and fibroblasts. Keratinocytes are the predominant keratin-producing cell in the epidermis, which protects the dermal layer of the skin via re-epithelialisation [183]. In the presence of a wound, the basal keratinocytes migrate from the wound edge and dermal appendages followed by cell proliferation that covers the wound surface [184]. A suboptimal level of ROS is essential to preserve the normal physiological function of keratinocytes in the skin’s epidermal layer. However, excessive accumulation of ROS leads to oxidative stress in the cells, thus resulting in the flattening of the dermal-epidermal junction and decrease skin barrier’s functionality [185]. Therefore, in the case of a hard-to-heal wound with an overproduction of ROS, alternative antioxidant agents from the treatments are essential for skin re-epithelialisation. On the other hand, dermal fibroblasts are responsible for forming an extracellular matrix during wound healing by synthesizing collagen and other proteins such as elastin and fibronectin, which assist in skin contraction [186]. Fibroblasts also produce matrix metalloproteinases that influence the proliferation of the keratinocytes during wound re-epithelialisation. The functionality of the fibroblast may be reduced due to the imbalance of cellular redox homeostasis because of the elevation of ROS level and oxidation stress. This could be restored with the presence of antioxidant constituents [187]. 

To assess the antioxidant ability of the materials, oxidative stress can be induced in the cell culture through various methods. Exposure to a high concentration of H_2_O_2_ is one of the most common methods of oxidative stress induction in cellular studies. In a low concentration, cellular antioxidant defense upregulates the expression of antioxidant enzymes such as peroxiredoxins and GSH-Px to eliminate H_2_O_2_, which controls the H_2_O_2_ concentration gradient and prevents the onset of oxidative stress [188]. However, a higher dose of H_2_O_2_ resulted in the collapse of the oxidation cycle of the enzymes due to hyperoxidation. In a study, Shi et al. administered a wide range of H_2_O_2_ concentrations (50–1000 µmol L^−1^) to induce oxidative stress in human skin fibroblasts, and 100 µmol L^−1^ was selected as the most ideal concentration for oxidative stress modelling [189]. Meanwhile, O’Toole et al. (1996) suggested that the concentration of H_2_O_2_ for keratinocytes should be 200 µM, which resulted in a 60–70% decrease in the proliferation rate [190]. Besides the exposure to radicals such as H_2_O_2_, UV exposure can also induce oxidative stress in the cell model by altering the cellular protein function by interacting with the aromatic amino acids. The UV radiation in a cell study could mimic the oxidative damage caused by sun exposure. Although there are many types of UV radiation, UVA and UVB remain the most common UV radiations used in experimental studies to induce oxidative stress. For example, Svoboda et al. applied UVA irradiation with a dose of 20 J cm^−2^, which inhibits the proliferation of keratinocytes [191]. Meanwhile, Vostalova et al. proposed a UVB irradiation at a dose of 200 mJ cm^−2^ in reducing caspase-3 activity in the keratinocytes [192].

**Table 4:** The in vitro studies of antioxidant biomaterials.

Type of Cells	Antioxidant Compound	Biomaterial	Assay	Outcomes	References
NIH-3T3 fibroblast cells	Gallic acid	Hexanoyl glycol chitosan	Live/Dead assay	The cell viability is more than 90% when incorporated in the hydrogel	[122]
	Chitosan	Hyaluronic acid	CCK-8 assay	The CCK-8 assay reported 100% cell viability after 24 h incubation and wound size decreased by 25.9% after 24 h	[145]
			Scratch assay		
	Catechol moiety	Nanofibrous mat	MTT	The incorporation of catechol increases the cell adhesion and viability by 100% and complete wound closure after 24 h	[132]
			Calcein AM		
			SEM		
			Scratch assay		
	Curcumin	Polyvinyl alcohol and alginate	MTT	The biomaterial shown to have more than 100% cell viability	[146]
	Vitamin K3-Carnosine peptide	Silk fibroin	Immunocytochemistry	Immunocytochemistry reported that the cells are actively proliferating when treated. The incorporation of bioactive molecules significantly increases the cell migration	[131]
			Scratch assay		
			Live/Dead assay		
	Honey	Ethylcellulose/gum tragacanth	MTT	The cell viability is more than 100% at day 7 for treated group and viability increased with increasing concentration of honey	[170]
	Phenolic acid	Polymer foams	Alamar blue	More than 79% of cell viable after 24 h incubation	[193]
	Propenoic acid	Gelatin	Live cell tracker	More than 90% reported in the cell viability and scratch area within 24 h.	[150]
			MTT		
			Scratch assay		
	Olives leaves *Camellia sinensis* extract	Carboxymethylcellulose	MTT	The cell viability decreases by 88% when the concentration increased to 100 µg/mL. The skin sensitization and irritation shown to have negative results	[151]
			h-CLAT assay		
			OECD 439 assay		
	Quinone	Silk-fibroin	MTT	The addition of quinone increased the cell viability by 100% after 6 days incubation and enhance the cell migration	[159]
			Scratch assay		
Human skin fibroblast cells	5-Hydroxymethylfurfural	Polyvinyl alcohol and sodium alginate	CCK-8 assay	Integration of 5-HMF does not give significant results in cell proliferation. However, biomolecule affect the migration rate at concentration 50.4 and 75.6 µg/mL at 60% and 80% healing rate, respectively and slightly increase the production of collagen	[123]
			CFSE		
			Scratch assay		
			Hydroxyproline assay (Collagen production)		
	Curcumin	Polyvinyl alcohol and alginate	MTT	The biomaterial is non-toxic and able to increase the proliferation rate by 120%	[146]
EA.hy926 keratinocyte cells	Catechol moiety	3-4-dihydroxyphenylalanine (DOPA)	MTT	DOPA integration shows higher cell viability and cell adhesion through MTT as well as rigorous proliferation, adhesion and network formed shown via SEM and complete wound scratch closure within 24 h and accelerate tube formation at 72 h	[132]
			Calcein AM		
			SEM		
			Scratch assay		
			RNA isolation		
			Immunocytochemistry		
			Tube formation assay		
	Wheat grass	Collagen	MTT	Increase proliferation rate by 120% in 24 h	[163]
L929 fibroblast cells	Ferulic acid	Polyvinyl alcohol and chitosan	MTT	6.25% integration of Ferulic acid showed 99% cell viability via MTT and 100% healed wound scratch by 24 h	[154]
			Scratch assay		
	Chitosan	Chitosan-sulfonamide	MTT	Retain cell viability higher than 70% after 72 h	[111]
	Humic acid	Alginate	MTT	Integration of humic acid show 8.18% higher cell viability of 100.80% compared to alginate alone	[149]
	Tannic acid	Hyaluronic acid	MTT	Low dose (250 µg mL^−1^) TA attain cell viability above 80%, as well for the love and dead assay, however higher dosage of AgNP (4 mM) incorporation showed significant reduce of cell viability (approx. 60%)	[152]
			Live/Dead assay		
Human umbilical vein endothelial cells (HUVEC)	*Gundelia tournefortii*	Silver nanoparticles	MTT	AgNP/GT IC_50_: 100 µg mL^−1^ AgNO3 IC_50_: 896 µg mL^−1^	[162]
	Quercetin	Polycaprolactone and gelatin	CCK-8 assay	CCK-8 report show P/Qu/Cup composites significantly enhanced cell proliferation whereas wound scratch assay show the composites are able to stimulate cell migration at 12 h.	[157]
			Scratch assay		

#### 6.1.2. Animal Studies

Typically, animal studies are vital for research studies as it helps to understand complex inquiries of disease progression, genetics, risk, or further biological mechanisms of a whole living system that would be technically impractical or unethical to be performed in human subjects. Rodents, such as bred rats and transgenic mice, are the most used animals in biomedical research [194]. The advantages of using animal models include the accuracy in studying the effects of biomaterials in the complex models, especially the antioxidant defense or response when exposed to oxidative stress [195]. The efficiency of the antioxidants in the biomaterials and the side effects of antioxidant constituents in the biomaterials should be determined before these antioxidant biomaterials can be clinically relevant for the downstream translational application. Numerous data can be obtained through in vivo models, including the wound healing ability, angiogenic properties, protein expression, antioxidant markers, oxidative stress assessment, and ROS scavenging activity [196]. The in vivo studies of various antioxidant biomaterials for wound healing purposes are summarised in Table 5.

Many types of wounds can be inflicted on the animal model to study the healing effects including burn, full-thickness, and diabetic wounds. The animals can be induced with diabetes via streptozotocin (STZ) administration, whereas a full-thickness cutaneous wound can be created using a biopsy punch. A burn wound can also be created by exposing the rodent’s skin to high-pressure steam [109,111,145]. Two of the most widely used wound analysis are wound closure analysis and haematoxylin-eosin (H&E) staining. The wound closure is analysed through photographic analysis (wound size) and analytical data of the wound measurement, whereas H&E staining is used to identify different types of tissues and their associated morphological changes [111,197]. 

#### 6.1.3. Clinical Studies

Wound treatment with antioxidant biomaterials has been found to be a suitable therapeutic alternative to achieve wound activation for non-healing or chronic wounds. As interest in the use of antioxidants continues to grow, clinical studies that involve human subjects are mandatory to validate those scaffolds that have shown efficacy in promoting in vitro and in vivo wound healing. The antioxidant dressing has been tested in an acute wound model in pigs with promising results [198]. However, chronic wounds would be the ideal target for this treatment. This is because chronic wounds are usually presented with oxidative stress, which arrests the wound in the inflammatory phase and prevents its progression into the other healing phases [199]. Recently, a new therapeutic approach through antioxidants in conjunction with naturally derived biomaterials has been applied in clinical trials towards patients suffering from non-healing and chronic wounds. Depending on the type of antioxidants and biomaterials used, they may either function as a skin equivalent or serve as a temporary wound cover or dressing [200]. However, data on the use of antioxidant biomaterials and human safety remains scarce. 

Based on the literature search, we identified several antioxidant compounds currently undergoing human clinical trials for wound therapies, which are summarised in Table 6. These antioxidant compounds demonstrated their potential efficacy as a standard wound healing treatment with supporting evidence from clinical studies. Most of the patients included in the study presented with chronic, full-thickness wounds, and severe comorbidities [201,202]. Hence, some patients could not finish the entire eight-week treatment period with the antioxidant dressings. In these studies, all patients were handled with good care during the treatment and clinically proven wound healing effectiveness with antioxidant biomaterials treatment. The treatment showed a better performance for daily clinical practice and the dressings were very well tolerated by the patients [199]. Nonetheless, more clinical trials are required to explore the proper role of antioxidant compounds integrated within a biomaterial scaffold in the application of wound healing. 

The field of antioxidant-based biomaterials for wound healing therapies is expanding rapidly with time. However, these dressings are often hindered by the low number of clinical trials that can provide concrete evidence regarding their safety and healing efficacy. Interpretations of human clinical studies are complicated by the different types of wounds evaluated, treatment with multiple bioactive agents, and different administration routes [203]. Basically, chronic wound healing, especially in patients with diabetes, is a significant clinical problem that often requires surgical intervention. That is also the reason why clinical trials need to be performed on a large group of patients to obtain accurate data. Darwin and Tomic-Canic [204] discussed the challenges in both pre-clinical and clinical wound research that cause slow progress and development of efficacious therapies. One of the main challenges in every clinical study is patient recruitment. The recruitment rates are always tied together with the costs of the clinical trials, leading to a major obstacle. The future of clinical studies will expand rapidly alongside research and development, and a better elucidation or explanation of the role of antioxidants in wound healing therapy will be discussed in depth.

**Table 6:** Studies about the effect of antioxidant compounds as biomaterials on the wound healing process (clinical trials).

Compound	Carrier Type	Wound Type	Population	Results	Outcome	Reference
Chitosan	Dressing (not specified)	Chronic wounds (pressure ulcers, vascular ulcers, diabetic foot ulcers, and wounds with minor infections)	90 patients	**Wound area**: Significantly reduced in the test group (65.97 ± 4.48%) than in the control group (39.95 ± 4.48%). **Pain score**: In the test group was 1.12 ± 0.23 and 2.30 ± 0.23 in the control group **Wound depth**: Lower in the test group (0.30 ± 0.48 cm) than in the control group (0.54 ± 0.86 cm)	11 wounds had healed within 30 days	[201]
	Film and hydrocolloid dressing	Superficial wound	244 patients where 86 were treated with chitosan derivative film, and 84 with hydrocolloid dressing	**Wound area**: Day 13, the mean wound epithelialisation in the chitosan derivative film group was 99.17%, while 99.84% was in the hydrocolloid group. **Pain score**: Chitosan derivative film experienced more pain during the removal of the dressing, less exudate, and less odor than hydrocolloid group	No significant difference between the two groups; one, treated with a chitosan derivative film, and two, treated with hydrocolloid dressing	[205]
Curcumin and galactomannan	Biofilm	Chronic wounds (type II diabetes)	31 patients	**Wound area**: On the 12-weeks period, 16 over 31 wounds totally healed	Antioxidant wound treatment is a suitable therapeutic alternative and good performance for daily clinical practice.	[202]
Curcumin and *N*-Acetyl cysteine (NAC)	Dressing (not specified)	Acute and chronic wounds (venous leg ulcer, calciphylaxis ulcer, traumatic and dehisced wounds)	31 patients	**Wound area**: On the 8-week period, 9 wounds (29%) completely healed, of which 7 were acute (77.8%) and 2 were chronic (22.2%) **Other effect**: The remaining wounds (22) presented a significant improvement after treatment	Treatment with the antioxidant dressing was more marked in the first 4 weeks, that the dressing works well both with acute and chronic wounds	[199]
*Leptospermum* honey	Gel	Partial-thickness burn wounds (face)	7 patients	**Wound area**: Healing time ranged from 3 to 14 days (mean, 8.1 days) **Other effect**: Wound cultures revealed normal bacterial growth on days 1 and 7 for all patients	Active *Leptospermum* honey (ALH) improves outcomes in patients with partial-thickness burns by enhancing healing and re-epithelialisation rates, as well as by protecting against antibiotic-resistant microorganisms	[204]
Quercetin	Nano-hydrogel	Diabetic foot ulcer	56 patients	**Wound area**: Nano-hydrogel embedded with quercetin and oleic acid demonstrated complete wound healing in 1 month **Other effect**: Only two patients of 56 (3.6%) were unresponsive to the treatment after 3 months. No local recurrence was observed during the follow-up period	No record on the development of other adverse drug reactions such as topical skin allergy, skin discoloration, or keloid scars.	[205]

## 7. Conclusions

The incorporation of antioxidants into biomaterials offers significant potential for wound healing applications. To achieve effective antioxidant therapy, the antioxidant needs to be delivered to the correct oxidation species and be delivered directly to the targeted tissue. This review presents an overview of the common natural-based materials used for developing biomaterials (fabrication of scaffolds), explores the bioactive compounds with antioxidant properties, and examines the biological properties and the main outcomes of healing processes in injured tissues that have been reported in the literature. There are a variety of scaffolds that have been reported to enhance the regeneration of damaged skin tissues by scavenging the excessive free radicals that confer a potential insult for the normal cellular activity and tissue function. Antioxidant biomaterials represent an emerging solution to attenuate oxidative stress with a huge impact in the field of skin tissue engineering and regenerative medicine. They can be processed into different structures and shapes, such as hydrogels, nanofibers, films/membranes, sponges, and nanoparticles, to further enrich their capability for tissue regeneration.

Scientists and researchers have the opportunity to explore different biomaterials that provide more active compounds with antioxidant properties and the potential for use as wound therapies. Future works could explore different biomaterials or scaffolds for wound healing applications and tissue regeneration. Another part that should be focused on is the application of different scaffolds and their sterile packaging before implantation onto a patient’s wound. At the same time, we also suggest the use of biodegradable and eco-friendly materials to overcome the problem of pollution issues and reduce the production of waste. 

## Figures and Tables

**Table 3:** The antioxidant assays of the biomaterials for wound healing application.

Antioxidant Assay	Antioxidant Component	Biomaterial	Type of Scaffold	Outcomes	References
DPPH	*Sarasinula marginata* extract	Natural rubber latex	Membrane	8.53 µg mL^−1^ *Sarasinula marginata* extract able to achieve more than 50% inhibition of radical when compared to ascorbic acid.	[74]
	Chitosan Curcumin	Hyaluronic acid and chitosan	Hydrogel	Concentration of 1–100 µg of curcumin gradually increase the scavenging activity by 33.6% to 84.6%.	[145]
	Catechol moiety	Not stated	Nanofibre	The bioactive nanofibrous mat manage to scavenge 50% of DPPH while the native construct only achieves 12.5% and 5%.	[132]
	Ferulic acid	Polyvinyl alcohol and chitosan	Film	Increasing concentration of bioactive molecules, increase the free radical scavenging activity with maximum value of 97.2% for 500 µL ferulic acid	[154]
	*Plantago lanceolata* *Tagates patula* *Symphytum officinale* *Calendula officinalis* *Geum urbanum*	Chitosan	Film	The evaluation of DPPH shown that bioactive compound is 1.953 mg TE/g	[109]
	Honey Curcumin	Polyvinyl alcohol and cellulose acetate	Nanofibrous mat	The honey-curcumin integrated scaffolds able to achieve 93% free radical activity	[156]
	Acacia gum	Acacia gum polysaccharide	Hydrogel	The bioactive film exhibits 51.35% of DPPH free radical inhibition	[125]
	Star anise	Chitosan	Not stated	Star anise able to reach 100% inhibition of DPPH at concentration of 25 µg/mL	[161]
	Honey	Ethylcellulose/gum tragacanth	Nanofibre	Electrospun fibre incorporated with 20% honey have more than 60% at 9 h incubation	[170]
	*Cannabis sativa*	Collagen	Hydrogel	The highest scavenging activity is 67 mg/g of *cannabis sativa* which value at 47.20%	[126]
	*Gundelia tournefortii*	Not stated	Ointment	The scavenging activity of bioactive compound is similar with AgNP which is 376 µg/mL	[162]
	Papain Alginate	Keratin/alginate, Keratin/agar, and Keratin/gellan	Patches	Bioactive compound able to decrease the DPPH concentration by 30.55%	[155]
	Propenoic acid	Gelatin	Hydrogel	The radical scavenging activity of biomaterials at 50% with concentration of 175 µg and 350 µg	[150]
	Curcumin	Chitosan-alginate	Nanofibre	Curcumin encapsulated biomaterial able to inhibit 41.37% DPPH at 60 µg/mL	[112]
	Olive leaves *Camellia sinensis* extract	Carboxymethylcellulose	Hydrogel	Incorporation of bioactive compound in hydrogel exhibit 83% radical scavenging activity	[151]
	Tannic acid	Hyaluronic acid	Hydrogel	The presence of tannic acid able to inhibit 80% of DPPH	[152]
	*Hypericum perforatum* oil	Chitosan	Cryogel	The scavenging activity increased to 53.2% when bioactive compound integrated into bioscaffold	[164]
	Quinone	Silk-fibroin	Nanofibre	The antioxidant activity resulted in increased IC50% at 5.5 µg of quinone incorporated in nanofibre	[159]
ABTS	Ferulic acid	Chitosan-alginate	Nanofibre	The presence of ferulic acid able to reduce ABTS more than 1.5 time of native biofilm	[112]
	*Plantago lanceolata* *Tagates patula* *Symphytum officinale* *Calendula officinalis* *Geum urbanum*	Chitosan	Film	The bio-composite result in ABTS value of 1.745 mg TE/g	[109]
	Olive leaves *Camellia sinensis* extract	Carboxymethylcellulose	Hydrogel	The compound result in 83% ABTS radical	[151]
DCFH-DA	Lignin	Lignin	Nanogel	Lignin able to reduce the intensity of DCF fluorescence signal indicate reduction of intracellular ROS	[62]
	Curcumin Chitosan	Hyaluronic acid and chitosan	Hydrogel	The treatment group decrease the fluorescence intensity with concentration as low as 1 µg	[145]
	Vitamin E	Not stated	Hydrogel	Significant decrease in DCF fluorescence intensity with treatment group of 600 µg/mL	[148]
	Tannic acid	Phenylboronic acid-modified hyaluronic acid	Hydrogel	Treatment group is significantly low DCF fluorescence intensity	[152]
Total antioxidant status kit	Humic acid Alginate	Alginate	Hydrogel	Dual compounds show higher antioxidant capacity, which is 0.25 nM compared to alginate composite, 0.22 nM	[149]
Lipid peroxidation	Quercetin	Collagen	Film	The absorbance of AIBN increases when quercetin was added	[153]

**Table 5:** The in vivo studies of antioxidant biomaterials for wound healing application.

Wound Type	Antioxidant Biomaterial	Parameter	Animal Model	Outcomes	References
Burn wound	Lignin nanogel	Wound closure, H&E, Ki67	Balb/c mice	**Wound closure**: Accelerate recovery. **Ki67**: protein increased and accelerate cell proliferation were observed. **H&E**: Low inflammatory cells in the treated wound, hair follicles and epithelium regeneration were observed.	[62]
	Chitosan films	Wound closure, H&E	Wistar rats	**Wound closure:** vascularisation and improved wound, formation of new epithelial layer on day 14. **H&E**: complete re-epithelialisation, mature epidermal, reduction of inflammatory infiltrate and congestion.	[154]
Full-thickness wound	Quercetin films	Wound closure, hydroxyproline, uronic acid, total protein, superoxide dismutase, catalase	Albino Wistar rats	**Wound closure**: 20% wound contraction; increase hydroxy proline (1.836 mg/100 mg tissue) and protein (76 mg/g tissue) content compared to control.	[153]
	Gallic acid hydrogel	Wound closure, Growth factor expression, H&E	Balb/c mice	**H&E**: 20% higher wound contraction (Day 10); wound completely heal (Day 15); 60 μm thicker granulation tissue **Growth factor expression**: High expression of TGF-β 13.6-; EGF 5.5-; VEGF fold on day 5, day 10 (6.3-fold) and day 15 (2.7-fold)	[122]
	5-hydroxymethylfurfural hydrogel	Wound closure, H&E, IHC	Sprague Dawley rats	**Wound closure**: Higher rate of wound closure **H&E**: Higher collagen disposition, wound tissue structure almost similar to healthy tissue. **IHC**: Significantly high VEGF expression and blood vessel formation compared to other groups.	[123]
	Catechol moiety nanofibre	Wound closure, H&E, IHC	Wistar rats	**Wound closure**: Complete wound closure (day 20) compared to control (70%). Significantly low granulation tissue and proliferation of fibroblastic. **H&E**: Show uniform collagen bundles, complete re-epithelialisation and expression of cytokeratin on day 20	[132]
	Curcumin hydrogels	Wound closure, Hydroxyproline, H&E, SEM	Wistar albino rats	**Wound closure**: wound shrinkage, 50% wound closure at day 16 **H&E**: No visible inflammation or fibrinoid. Show re-epithelialisation and rapid hair growth **SEM**: Show newly formed collagen and fibroblast in the epidermal	[146]
	Flaxseed gum hydrogels	Wound closure	C57BL/6 mice	**Wound closure**: (Day 10) Capillaries formation, differentiated collagen fibres, clear tissue structure, rapid hair follicles proliferation.	[124]
	*Madhuca longifolia* seed nanoparticles	Wound closure, H&E	Swiss albino mice	**Wound closure**: Faster wound closure rate (80.33%) and wound epithelisation (18 days) compared to the controls. **H&E**: Stratum corneum recover and rapid hair growth	[124]
	Acacia gum hydrogel	H&E	Balb/c mice	**H&E**: (Day 12) High collagen production, vessel formation and negligible inflammation	[125]
	Wheat grass aerogel	Wound closure	Wistar rats	**Wound closure**: (DAY 9) 75% wound reduction, significantly high rate of wound size reduction compared to other groups. Healed wound were observed on day 18.	[163]
	*Gundelia tournefortii* nanoparticles	Wound closure, H&E	Albino mice	**Wound closure**: Significantly decreased wound area compared to other groups. **H&E**: significantly reduced neutrophils and lymphocytes, high formation of blood vessels, fibrocytes, fibroblast, hydroxyproline, hexosamine and hexuronic acid compared to other groups.	[162]
	Konjac glucomannan- EGCG films	Wound closure, H&E	Sprague Dawley rats	**Wound closure**: Accelerate wound closure and healed wound on day 13. **H&E**: formation of keratin and epidermis layer, neovascularisation, blood vessels formation and mature hair follicles were observed.	[160]
	Propenoic acid hydrogels	Wound closure, H&E	Wistar rats	**Wound closure**: (Day 16) Complete wound closure. **H&E**: Significant wound closure and hair follicle growth were observed.	[150]
	Curcumin bioactive glass	Wound closure, H&E, Hydroxyproline	Wistar rats	**Wound closure**: (Day 14) complete epidermis re-epithelialisation. **Hydroxyproline**: Increase hydroxyproline content (33.5%) compared to other groups.	[165]
Diabetic wound	Chitosan-hyaluronic acid hydrogels	Wound closure, H&E	C57BL/6 mice	**Wound closure**: Healing rate of 54.5%, (Day 10) 88.3% wound closure were observed. **H&E**: Abundant tissue granulation, compact and layered.	[145]
	*Plantago lanceolata, Tagates patula, Symphytum officinale, Calendula officinalis, Geum urbanum* loaded chitosan films	Wound closure, H&E	Wistar rats	**Wound closure**: (Day 14) treated wound 97.47% healed **H&E**: rapid blood vessels proliferation, collagen fibres formation and fibroblast proliferation were observed.	[109]
	Vitamin K3- Carnosine peptide electrospun fibre	Wound closure, H&E	Sprague Dawley rats	**Wound closure**: wound closure rate (Day 3–9) 55.05 ± 10.2, 78.12 ± 14.5, 100% **H&E**: low number of inflammatory cells observed in the treated wound.	[131]

## Data Availability

The data presented in this study are available on request from the corresponding author.
